# Synergistic Effects of Age on Patterns of White and Gray Matter Volume across Childhood and Adolescence^1^,^2^,^3^

**DOI:** 10.1523/ENEURO.0003-15.2015

**Published:** 2015-07-13

**Authors:** Signe Bray, Mark Krongold, Cassandra Cooper, Catherine Lebel

**Affiliations:** 1 Department of Radiology, Cumming School of Medicine, University of Calgary, Calgary, Alberta, Canada T2N 1N4; 2 Department of Pediatrics, Cumming School of Medicine, University of Calgary, Calgary, Alberta, Canada T2N 1N4; 3 Child and Adolescent Imaging Research Program, Alberta Children's Hospital, Calgary, Alberta, Canada T3B 6A8; 4 Alberta Children’s Hospital Research Institute, Calgary, Alberta, Canada T3B 6A8; 5 Biomedical Engineering Graduate Program, University of Calgary, Calgary, Alberta, Canada T2N 1N4

**Keywords:** cortex, MRI, structural covariance network, white matter

## Abstract

The human brain develops with a nonlinear contraction of gray matter across late childhood and adolescence with a concomitant increase in white matter volume. Across the adult population, properties of cortical gray matter covary within networks that may represent organizational units for development and degeneration. Although gray matter covariance may be strongest within structurally connected networks, the relationship to volume changes in white matter remains poorly characterized. In the present study we examined age-related trends in white and gray matter volume using T1-weighted MR images from 360 human participants from the NIH MRI study of Normal Brain Development. Images were processed through a voxel-based morphometry pipeline. Linear effects of age on white and gray matter volume were modeled within four age bins, spanning 4-18 years, each including 90 participants (45 male). White and gray matter age-slope maps were separately entered into *k*-means clustering to identify regions with similar age-related variability across the four age bins. Four white matter clusters were identified, each with a dominant direction of underlying fibers: anterior–posterior, left–right, and two clusters with superior–inferior directions. Corresponding, spatially proximal, gray matter clusters encompassed largely cerebellar, fronto-insular, posterior, and sensorimotor regions, respectively. Pairs of gray and white matter clusters followed parallel slope trajectories, with white matter changes generally positive from 8 years onward (indicating volume increases) and gray matter negative (decreases). As developmental disorders likely target networks rather than individual regions, characterizing typical coordination of white and gray matter development can provide a normative benchmark for understanding atypical development.

## Significance Statement

The structure of the brain changes across late childhood and adolescence: gray matter volume decreases and white matter volume increases. Gray matter changes occur within networks that may be targets for neurodegenerative, developmental, and psychiatric disorders. This study demonstrates that changes in white matter volume are also coordinated across regions, and that changes in these clusters parallel corresponding gray matter clusters. While gray matter clusters show a posterior to anterior organization, we observe here that white matter volume groups into regions with similar fiber orientation. This work adds to our understanding of typical gray and white matter development, which ultimately can help understand how the brain may be developing abnormally in neurodevelopmental disorders.

## Introduction

As the brain develops across late childhood and adolescence, a pattern of white matter expansion (Giedd et al., 1999; Paus et al., 1999; Sowell et al., 2002; Taki et al., 2013) and gray matter contraction (Sowell et al., 2003, 2004; Gogtay et al., 2004; Shaw et al., 2008) has been observed. These co-occurring phenomena are widely considered to be the product of developmental exuberance (Innocenti and Price, 2005), through which an overproduction of connections is followed by a selection process. White matter volume expansion is thought to reflect both an increase in myelination and axonal diameter (Yakovlev and Lecours, 1967; Benes, 1989; Benes et al., 1994; Rademacher et al., 1999; Paus, 2010). Observed patterns of gray matter thinning may reflect synaptic pruning (Huttenlocher, 1979), changes in size and number of glia or size of neurons (Elgeti et al., 1976; Drevets et al., 1998; Cotter et al., 2002), vasculature (Vaidya et al., 2007), or changes in myelination of superficial white matter (Sowell et al., 2004; Shaw et al., 2008, but see Wu et al., 2014).

Distributed cortical regions show correlated anatomical features across the population (Mechelli et al., 2005; Lerch et al., 2006; Chen et al., 2008; Tijms et al., 2012; Alexander-Bloch et al., 2013b; Evans, 2013) in networks similar to those defined by resting state functional connectivity (Segall et al., 2012; Alexander-Bloch et al., 2013b) and white matter tractography (Gong et al., 2012). These findings have been extended to describe coordinated cortical development across childhood and adolescence (Zielinski et al., 2010; Raznahan et al., 2011b; Alexander-Bloch et al., 2013b, 2014; Khundrakpam et al., 2013). The importance of these findings is underscored by the suggestion that neurodegenerative, psychiatric, and neurodevelopmental disorders may target cortical networks rather than specific regions (Seeley et al., 2009; Raznahan et al., 2010; Reid et al., 2010; Zielinski et al., 2012; Alexander-Bloch et al., 2014).

White matter tracts also show developmental changes in structural properties (Barnea-Goraly et al., 2005; Ben Bashat et al., 2005; Giorgio et al., 2008; Lebel and Beaulieu, 2011). Fractional anisotropy (FA), a measure of coherent fiber orientation linked to myelination and axon packing (Beaulieu, 2002), increases in most tracts (Barnea-Goraly et al., 2005; Lebel et al., 2008) and peaks in early adulthood before declining (Lebel et al., 2012). Mean diffusivity (MD), a measure reflecting water content and density, shows an opposite pattern, declining across adolescence and increasing in adulthood (Lebel and Beaulieu, 2011). The volume of white matter tracts also typically increases across childhood, though the relationship between tract-volume and microstructural parameters is complex (Lebel and Beaulieu, 2011).

Although gray matter developmental networks are increasingly well characterized, the relationship between white matter structural changes and network-level gray matter development remains unclear. In the present study, we tested the hypothesis that clusters of white matter regions would show coordinated volume development, parallel to gray matter clusters. That is, clusters of white matter regions showing coordinated variability with age (e.g., volume expansion) would be inversely associated with changes in gray matter volume (e.g., contraction), in related regions, across childhood and adolescence.

## Materials and Methods

### Participants and neuroimaging data

Neuroimaging data were obtained from the NIH MRI study of Normal Brain Development's Pediatric MRI Data Repository (Evans and Brain Development Cooperative Group, 2006). The cohort includes 433 typically developing participants, male and female, aged 4:6-18:3 years. All subjects are purported to be normal and healthy, e.g., no history of brain disease or trauma, with an IQ > 70. Analyses reported here used T1-weighted images collected on 1.5 T MRI scanners (GE or Siemens) at six sites (Boston Children's Hospital; Cincinnati Children's Hospital Medical Center; University of Texas Houston Medical School; Neuropsychiatric Institute and Hospital, UCLA; Children's Hospital of Philadelphia; and Washington University, St. Louis). Parameters for whole-brain T1-weighted acquisitions were standardized across sites: 3D RF-spoiled gradient echo, TR = 22-25 ms, TE = 10-11 ms, sagittal acquisition, FOV = AP 256, LR 160-180. Resolution was typically 1 mm^3^; however, on GE scanners on which thickness was increased up to 1.5 mm and in some participants resolution was decreased to 3 mm^3^ to enable more rapid imaging. For our sample we generated four evenly sized groups of participants (90) with an equal number of males and females (45), for a total sample including 360 high-quality scans. Age groups were 4-8, 8-10.5, 10.5-13.5, and 13.5-18.5 years; detailed information about participants is provided in Table 1.

**Table 1.** Participant demographics

	**Group 1**	**Group 2**	**Group 3**	**Group 4**
Mean	6.47(0.81)	9.16(0.76)	12.04(0.84)	16.03(1.27)
Age range (years)	4.80-7.85	8.02-10.47	10.68-13.50	13.99-18.35
Mean IQ (SD)	110.9(16.0)	112.5(12.9)	112.1(10.7)	109.0(11.4)
IQ range	79-156	77-160	84-131	78-132
Handedness R:L	83:7	80:10	80:10	81:9
Gender M:F	45:45	45:45	45:45	45:45
Mean adjusted income Thousands (SD)	71.1(32.5)	70.0(30.6)	70.1(32.0)	70.0(31.4)

### VBM processing

T1-weighted MRI scans were processed through a voxel-based morphometry (VBM) pipeline in SPM12b. Steps included segmentation and normalization using a custom template generated with the DARTEL Toolbox (Ashburner, 2007). Normalized gray and white matter segmented images were modulated to “preserve amounts” and smoothed using an 8 mm Gaussian kernel. All segmentations were visually inspected prior to analysis. VBM tools were also used to identify potential outliers by calculating the squared distance to sample mean in each age bin; no outliers were identified in this step.

### Linear age models

As developmental changes in gray matter volume across childhood and adolescence are known to be nonlinear (Gogtay et al., 2004; Shaw et al., 2008; Raznahan et al., 2011a), our sample was divided into four age bins similar to Zielinski et al. (2010) and Khundrakpam et al. (2013). Two general linear models were estimated in each age group, modeling a linear effect of mean-centered age on gray and white matter volume separately. Models included effects of gender, site (one regressor per site), and a linear effect of image resolution. Explicit masks were used to spatially constrain the analyses; gray and white matter masks were created using the Masking Toolbox in SPM12b (Ridgway et al., 2009) and constrained to probabilities >0.4 to ensure that there was no overlap in gray and white matter masks. Neither proportional scaling nor total brain volume regression were used in the main models reported here. However, both methods were tested in additional analyses, as described below.

### Clustering based on gray and white matter age slope

For each tissue type (white and gray matter), parameter estimates for the effect of age in each of the four age bins were obtained for each voxel. All parameter estimates (β-values) were entered into a pair of matrices, one gray and one white matter, in which each row corresponded to a voxel and each column corresponded to an age bin. Analyses were performed on all voxels independent of the significance of age effects, that is, voxel-level significance of age effects was not assessed as part of this study. Matrices were entered into *k*-means clustering in MATLAB to identify clusters of voxels with similar age slopes across these developmental stages. Clustering was seeded with random centers and repeated 10 times; 2-10 cluster solutions were tested, and peak silhouette values (Kaufman and Rousseeuw, 1990) were used to identify the optimal clustering solution for each tissue class.

### Directional bias in white matter clusters

Visual inspection of white matter clusters indicated a potential directional bias. To test this, white matter clusters were compared against directional maps from the ICBM-DTI-81 Atlas (Mori et al., 2008) to determine the primary direction of white matter fibers in each cluster. This atlas includes estimated eigenvalues for eigenvectors corresponding to three principal directions (*x*: right–left, *y*: anterior–posterior, *z*: superior–inferior). To determine whether voxels assigned to each white matter cluster had a preferred direction, the three eigenvalue maps were masked by each white matter cluster. For each cluster the subset of voxels with a dominant orientation along one of these principal directions was obtained by thresholding to include only voxels for which at least one eigenvalue was ≥0.4. We then calculated the proportion of voxels for which the maximum eigenvalue was in each principal direction.

### Effects of site and resolution on VBM segmentation

As previous studies have shown that VBM segmentations may be affected by data collection site and acquisition parameters (Pardoe et al., 2008; Pereira et al., 2008; Focke et al., 2011; Takao et al., 2013), additional analyses were run to investigate effects of data collection site and image resolution. We note that participant age did not significantly vary by site (*F*_(1,360)_ = 0.02, *p* = 0.88). Resolution did show a significant negative trend with age as the youngest participants were more likely to have larger voxel size (*F*_(1,360)_ = 12.6, *p* < 0.001). However, resolution did not significantly vary with age in individual age bins, though a trend remained in the youngest bin (*p* = 0.051, *p* = 0.73, *p* = 0.60, *p* = 0.22). To determine which regions may be affected by these parameters, models were run for gray and white matter volume separately including all 360 participants; effects of age, resolution, gender, and site were modeled (one column per site). *F*-contrasts were used to identify regions showing linear effects of resolution on gray and white matter volume, and nonlinear effects of site. We then compared clustering results, for models that included these covariates with a set of models that did not include site and resolution covariates, to assess effects of these parameters on clustering results.

### Effects of modeling total gray and white matter volume

Many VBM studies model effects of total tissue volume, enabling the identification of regions that discriminate between groups after differences in total volume are accounted for (Peelle et al., 2012). Significance of regional effects of age is sensitive to the choice of model (Peelle et al., 2012). For the main analysis here, we chose not to account for total gray and white matter volume, as our goal was simply to model age trends and not to identify regions where age effects were greater than the mean. However, to investigate differences in our results when accounting for total volume, two additional models were run, using proportional scaling by total tissue volume and including total tissue volume as a nuisance covariate.

### Effects of age on image contrast

T1-image contrast is known to increase over the first few years of childhood (Paus et al., 2001; Shi et al., 2010). Although the population assessed here was older (i.e., the youngest participant was 4.8 years), systematic effects of image contrast may nonetheless contribute to variable quality of gray/white segmentation, and these effects may vary between brain regions. To assess effects of image contrast as a function of age, bilateral masks covering frontal, temporal, parietal, and occipital gray and white matter regions were generated using the Wake Forest Pick Atlas tool (Maldjian et al., 2003) and the TD-ICBM-152 atlas (Mazziotta et al., 2001). These masks were warped into each participant's native space and used to extract regional gray and white matter values. Contrast was calculated in each subject and each region as follows: *C* = (white matter intensity–gray matter intensity)/gray matter intensity. We then assessed effects of age and site on contrast across the sample and within each age bin.

## Results

**Figure 1.**
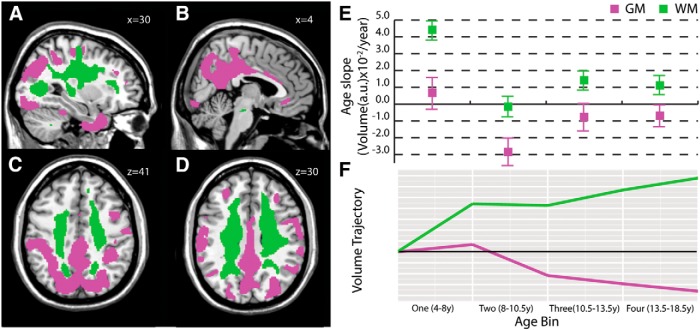
**Superior corona radiata/posterior gray matter clusters.** This white matter cluster included deep white matter of the superior longitudinal fasciculus, superior corona radiata, and body of the corpus callosum (***A***, ***C***, ***D***) and included mostly (68% of voxels) superior–inferior-oriented voxels. The corresponding gray matter cluster included primarily posterior cortical regions (***A-D***), including precuneus (***B***) and bilateral intraparietal sulcus (***C***). Mean gray and white matter slopes for the cluster with SDs (***E***) and a graphical illustration of volume trajectories (***F***) are shown for all four age bins. GM, gray matter; WM, white matter.

**Figure 2.**
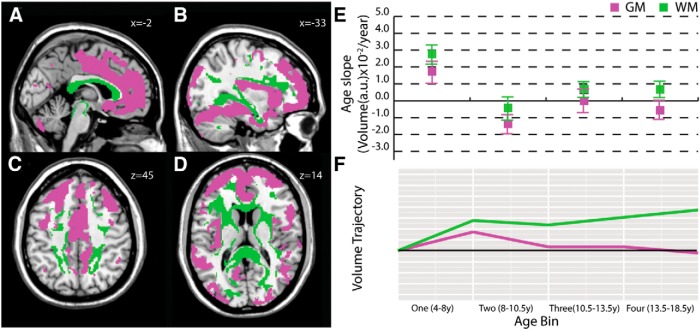
**Medial callosal white matter/anterior gray matter clusters.** This white matter cluster included medial corpus callosum (***A***), anterior internal capsule (***D***), and superior parietal lobule white matter (***C***) and was primarily ordered left–right (70%). The corresponding gray matter cluster included anterior cingulate and medial prefrontal cortex (***A***, ***B***, ***C***) and insular (***B***, ***D***) and temporal regions (***B***). Mean gray and white matter slopes for the cluster with SDs (***E***) and a graphical illustration of volume trajectories (***F***) are shown for all four age bins. GM, gray matter; WM, white matter.

**Figure 3.**
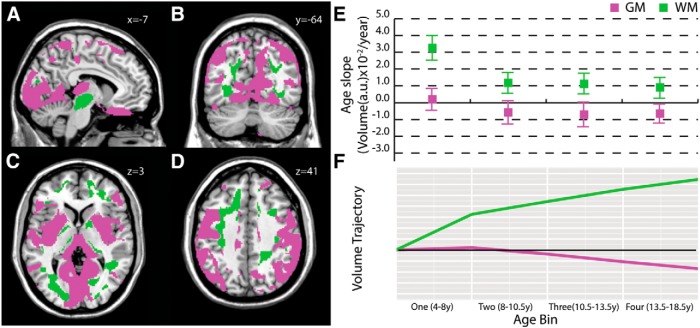
**Frontal and occipital white matter/visuomotor gray matter.** This white matter cluster included mostly superior–inferior-oriented voxels (74%) in superior cerebellar peduncle (***A***), occipital and superior parietal (***B***) and superior frontal gyrus white matter (***C***, ***D***), posterior thalamic radiation (***B***, ***C***), and precentral gyrus white matter (***D***). The corresponding gray matter cluster recruited cuneus (***A***, ***B***,***C***), motor (***D***), superior parietal (***B***), and lateral prefrontal (***C***) regions. Mean gray and white matter slopes for the cluster with SDs (***E***) and a graphical illustration of volume trajectories (***F***) are shown for all four age bins.

**Figure 4.**
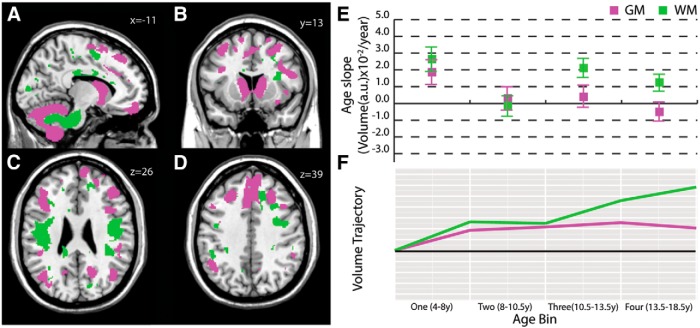
**Cerebellar white and gray matter clusters.** This white matter cluster included the cerebellum and cerebellar peduncles (***A***), including white matter in portions of the superior longitudinal fasciculus (***C***); voxels in this cluster were predominantly oriented anterior–posterior (58%). The corresponding gray matter cluster included the bilateral cerebellum (***A***), caudate (***B***), and dorsomedial prefrontal cortex (***D***). Mean gray and white matter slopes for the cluster with SDs (***E***) and a graphical illustration of volume trajectories (***F***) are shown for all four age bins.

White matter clusters^a^ showed a peak silhouette value at the four-cluster solution and gray matter clusters^b^ at the two-cluster solution (this solution divided cerebral cortex from cerebellum). As the goal of this study was to identify clusters of white matter regions with coordinated developmental patterns, in relation to gray matter clusters, both gray and white matter was divided into four clusters, which were subsequently paired based on adjacency of regions (Figs. 1-4). Gray and white matter structures were identified through visual inspection and comparison to gray and white matter atlases (Tzourio-Mazoyer et al., 2002; Oishi et al., 2011).

### Superior corona radiata white matter/precuneus and intraparietal sulcus (posterior) gray matter

**Figure 5.**
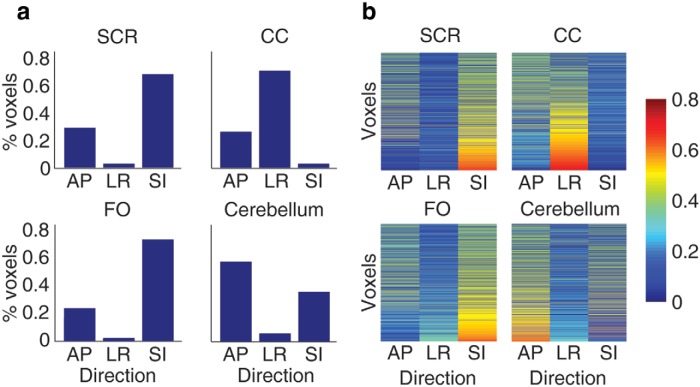
**Preferred white matter direction in each cluster.** For this analysis, the voxels within each cluster were thresholded to only those voxels with an eigenvalue ≥0.4 in one of the three canonical directions. ***a***, Shows the proportion of voxels for the maximum value in each direction. ***b***, Illustrates eigenvalues for all three directions across all voxels with at least one eigenvalue ≥0.4, sorted by maximum value in each row (i.e., each row is one voxel; heat map indicates the eigenvalue at that voxel for each canonical direction). SCR, superior corona radiata; CC, corpus callosum; FO, fronto-occipital; AP, anterior–posterior; LR, left–right; SI, superior–inferior.

One white matter cluster included the superior longitudinal fasciculus, superior corona radiata, and body of the corpus callosum (Fig. 1*A*,*C*,*D*), as well as a region along the posterior thalamic radiation (Fig. 1*C*). White matter voxels were predominantly (68%; Fig. 5) superior–inferior in orientation (Fig. 5). The most spatially similar gray matter cluster included primarily posterior cortical regions (Fig. 1*A-D*) such as the precuneus (Fig. 1*B*) and bilateral intraparietal sulcus (Fig. 1*C*). This cluster also included anterior temporal cortex (Fig. 1*A*) and smaller bilateral regions of posterior middle frontal gyrus (Fig. 1*D*). The gray matter cluster was characterized by a steep negative slope in the 8-10.5 year age bin and more positive slopes in other age groups; white matter age slopes followed a similar trend, though slopes were generally positive (indicating increasing volume with age; Fig. 1*E-F*).

### Medial corpus callosum white matter/anterior cingulate, prefrontal cortex, and insula (anterior) gray matter

A second white matter cluster (Fig. 2) included medial corpus callosum (Fig. 2*A*), anterior internal capsule (Fig. 2*D*), superior parietal lobule white matter (Fig. 2*C*), posterior thalamic radiation and retrolenticular portion of the internal capsule (Fig. 2*D*), and inferior frontal gyrus white matter (Fig. 2*B*). White matter voxels were mostly left–right oriented (70%; Fig. 5). The corresponding gray matter cluster included anterior cingulate and medial prefrontal cortex (Fig. 2*A-C*) and insular (Fig. 2*B*,*D*) and temporal regions (Fig. 2*B*). Gray matter age slopes (Fig. 2*E*,*F*) indicated the greatest volume decreases in the 8-10.5 year age bin, though slopes in all age bins were more moderate than in the posterior cluster (Fig. 1*E*,*F*). White matter slopes paralleled gray matter, but were generally positive, except for slight volume decreases in the 8-10.5 year age bin (Fig. 2*E*,*F*).

### Occipital, parietal, and prefrontal white matter/visual and motor gray matter

A third white matter cluster (Fig. 3) included superior cerebellar peduncle (Fig. 3*A*), occipital and superior parietal white matter (Fig. 3*B*), superior frontal gyrus white matter (Fig. 3*C*,*D*), posterior internal capsule (Fig. 3*C*), posterior thalamic radiation (Fig. 3*B*,*C*), and precentral gyrus white matter (Fig. 3*D*). This white matter cluster showed a strong superior–inferior orientation (74%; Fig. 5). The corresponding gray matter cluster involved cuneus (Fig. 3*A-C*), motor (Fig. 3*D*), superior parietal (Fig. 3*B*), and lateral prefrontal (Fig. 3*C*) regions. Among the identified gray matter clusters, this set of regions showed the most moderate slopes—slightly positive in the youngest age bin and relatively stable across the 8-18.5 year age range (Fig. 3*E*,*F*). White matter slope trajectories, again, followed a similar trend to gray matter, with a moderate but consistently positive slope from ages 8-18.5 years (Fig. 3*E*,*F*).

### Cerebellar peduncles/cerebellum

A fourth pair of gray and white matter clusters captured the cerebellum and cerebellar peduncles (Fig. 4). The white matter cluster included bilateral cerebellar peduncles (Fig. 4*A*) and portions of the superior longitudinal fasciculus (Fig. 4*C*); voxels were mainly anterior–posterior oriented (58%; Fig. 5). The gray matter cluster included bilateral cerebellum (Fig. 4*A*), but also caudate (Fig. 4*B*) and dorsomedial prefrontal cortex (Fig. 4*D*). For both gray and white matter, the slopes for this cluster were generally positive; white matter slope was slightly negative in the 8-10.5 year age bin and gray matter slopes became slightly negative in the 14-18.5 year age bin (Fig. 4*E*,*F*).

### Effects of data collection site and resolution

**Figure 6.**
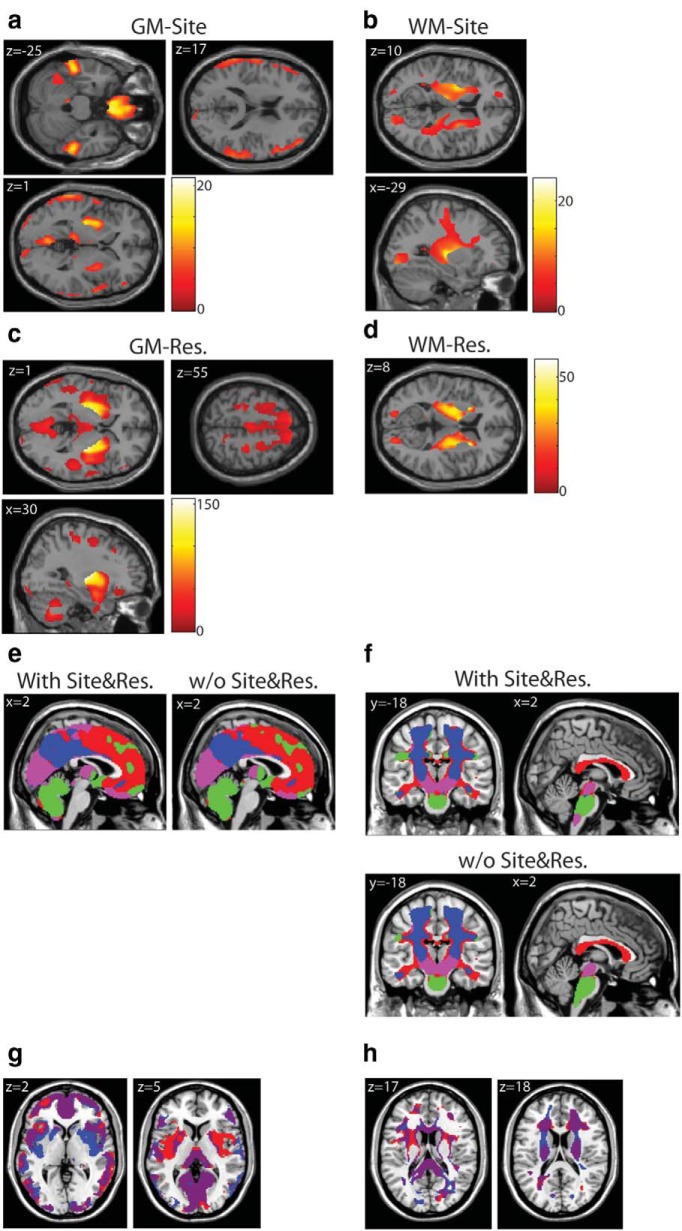
**Effects of site and resolution on regional volume and clustering results. *a***, ***b***, Results of an *F*-contrast for effects of site, thresholded at *p* < 0.001 uncorrected, on gray and white matter volume, respectively. Site effects were identified around the putamen and internal capsule as well as medial orbital and peripheral gray matter. ***c***, ***d***, Results of an *F*-contrast for effects of resolution, thresholded at *p* < 0.001 uncorrected, on gray and white matter volume, respectively. Effects were again concentrated around the internal capsule, with gray matter effects in dorsal prefrontal, occipital cortices, and cerebellum. ***e***, ***f***, Illustrate clustering results for age β-estimates from gray (***e***) and white (***f***) matter models that included effects of site and resolution (left and superior) and from models that did not include these effects (right and inferior). We note that these are largely similar. ***g***, ***h***, Illustrate regions of overlap (purple) and difference (red and blue) in cluster assignment for gray (**g**) and white (***h***) matter clusters when site and resolution are taken into account. The only substantial differences in clustering results were in posterior putamen and near the internal capsule. GM, gray matter; WM, white matter.

Previous studies have noted that VBM estimates of gray and white matter volume are sensitive to differences in MR scanner and image resolution (Pardoe et al., 2008; Pereira et al., 2008; Focke et al., 2011; Takao et al., 2013). As the present study made use of a multisite dataset, additional analyses were run to estimate potential impact of these factors on our results. A general linear model was estimated using the entire sample of 360 participants, including a linear effect of resolution and separate regressors modeling effects of each site. *F*-contrasts were used to identify regions sensitive to these effects. Results of *F*-contrasts for site are shown in Figure 6, *a* and *b*, thresholded at *p* < 0.001 uncorrected, for gray and white matter, respectively. We observe significant effects of site around the posterior putamen, orbitofrontal, inferior temporal, and peripheral gray matter in Figure 6*a*. Significant effects on white matter volume were most prominent around the internal capsule (Fig. 6*b*). Results of *F*-contrasts for resolution are shown in Figure 6, *c* and *d*, thresholded at *p* < 0.001 uncorrected, for gray and white matter, respectively. Affected gray matter regions were similarly concentrated around the posterior putamen and insula and occipital and dorsal prefrontal regions (Fig. 6*c*). For white matter, similar to effects of site, effects of resolution were largely concentrated around the internal capsule (Fig. 6*d*). We next compared clustering results for age β-values from models that did and did not include effects of site and resolution. These results are shown in Figure 6, *e* and *f*. We note that clustering results were largely similar between these two models. Figure 6, *g* and *h*, shows gray and white matter clusters obtained from these two models overlaid; regions of overlap are shown in purple. The only regions where cluster assignment substantially differed were around the putamen and internal capsule. Overall these results suggest that effects of site and resolution may have a fairly localized effect in subcortical regions, and we note that reliability of cluster assignment in these regions is a limitation of the present work.

### Effects of modeling total gray and white matter volume

**Figure 7.**
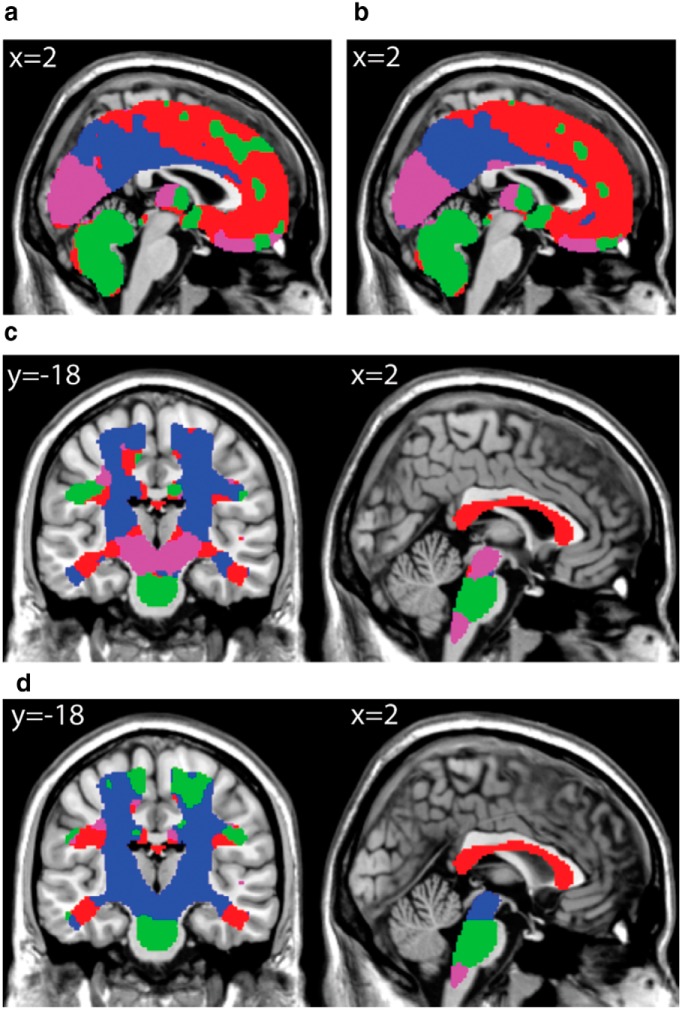
**Gray and white matter clusters derived from models accounting for total tissue volume. *a***, ***b***, Gray matter clusters for models using proportional scaling (***a***) and ANCOVA (***b***). ***c***, ***d***, White matter clusters for models using proportional scaling (***c***) and ANCOVA (***d***) models. Clusters are similar to Figure 6, *e* and *f*, with a notable difference in the white matter cluster in the midbrain for the ANCOVA model (***d***).

Two additional analyses were run using proportional scaling by total tissue volume and including total tissue volume as a covariate (ANCOVA). The resulting parameter estimates for age were entered into a similar cluster analysis as that described above. These analyses identified very similar clusters (Fig. 7), however, with some differences specifically in white matter clustering in the midbrain for the ANCOVA model.

### Effects of image contrast

White/gray matter contrast values for each lobe (frontal, temporal, parietal, and occipital) were entered into ANOVAs modeling effect of site with age as a covariate. Models were run across the entire sample and within each age bin. This analysis showed a significant effect of site in the temporal (*F*_(1,357)_ = 16.6, *p* < 0.001) lobe and a trend level effect in the frontal lobe (*F*_(1,357)_ = 4.3, *p* = 0.04 uncorrected for multiple comparisons). Over the entire sample there was a trend-level, negative association with age in the parietal cortex (*F*_(1,357)_ = 3.9, *p* = 0.048 uncorrected). However, age was not a significant predictor of contrast within any of the age bins, for any of the lobes. From these results we conclude that image contrast is unlikely to have biased age slopes in this analysis.

## Discussion

In this study, white and gray matter volumes were divided into clusters based on the similarity of age-related volume changes from 4-18 years. The four identified white matter clusters each showed a dominant orientation of fibers (anterior to posterior, left to right, and two clusters superior to inferior), and could be uniquely matched to a spatially proximal gray matter volume cluster. Gray matter clusters corresponded to cerebellar, medial/anterior, and sensorimotor clusters, respectively. Within gray and white matter network pairs, slopes followed similar trajectories across ages.

To our knowledge, this study is the first to investigate the organization of age-related structural variability in white matter volumes. Our results suggest that data-driven clustering of age-related variability in white matter volume can, to some extent, recover anterior-to-posterior, left-to-right, and superior-to-inferior directional components. While previous work has shown region- and tract-specific white matter volume changes with age (Tamnes et al., 2010; Lebel and Beaulieu, 2011), our results suggest a relationship between age-related variability in white matter volume and fiber direction. We also note that the identified clusters generally did not segregate deep from superficial white matter. These findings add to our understanding of properties of white matter volume development.

Furthermore, our results demonstrate a spatial and temporal relationship between patterns of age variability in white and gray matter volume. Previous work had shown that in individuals aged 8-30 years (Tamnes et al., 2010) there are negative correlations between cortical thickness and volumes of corresponding gyral white matter. Wu et al. (2014) found that the association between superficial white matter FA and cortical thickness was positive in unimodal sensory areas, but negative in polymodal regions. In adolescents, a negative correlation between gray matter density and FA in the right superior corona radiata has been described (Giorgio et al., 2008). These studies show that the general pattern of maturational contraction of gray matter is coupled with changes in white matter properties, including increased FA and volume. The present study builds on these findings by showing that there is a network organization in patterns of age-related variability in both gray and white matter volumes, and that these clusters are coupled based on both spatial proximity and similarity in age slopes.

White matter development across childhood and adolescence is characterized by increased FA and volume and reduced MD (Barnea-Goraly et al., 2005; Ben Bashat et al., 2005; Giorgio et al., 2008; Lebel and Beaulieu, 2011). These processes occur asynchronously across white matter regions (Lebel et al., 2008), and are believed to reflect changes in myelination and axonal packing (Yakovlev and Lecours, 1967; Beaulieu, 2002). White matter properties such as FA appear to be influenced by both genetic (Kochunov et al., 2015) and environmental (Hofstetter et al., 2013) factors. We speculate that genes expressed within regions or networks may contribute to coordinated patterns of volume development observed here. Though often considered separately, gray and white matter development may reflect processes occurring in the same cells (though changes in gray matter may also reflect changes in glial cells or vasculature; Zatorre et al., 2012). We further speculate that coordinated white matter expansion and gray matter contraction may reflect a synergistic process of increased myelination and decreased synaptic or dendritic density that occurs as networks mature. We note, however, that MRI studies are quite limited in resolution and thus have limited ability to test these hypotheses directly.

Different distributed networks have been identified in fMRI connectivity studies, including default-mode or task-negative, frontoparietal/dorsal attention/task positive, ventral attention, salience, visual, motor, and subcortical networks (Fox et al., 2005; Power et al., 2011). A previous seed-based study showed that longitudinal change in cortical thickness in core regions of the task-positive and task-negative networks are correlated (Raznahan et al., 2011b). We note that using a data-driven approach, the gray matter clusters identified here did not specifically resemble either of these two networks. Instead, we found a posterior cluster that included both precuneus and bilateral intraparietal sulcus, regions associated with both the task-positive and task-negative networks. An anterior cluster included both medial prefrontal task-negative regions and cingulate and dorsal prefrontal regions associated with task-positive and salience networks. A recent study using a qualitatively similar approach to that described here also identified one primarily parietal network and several networks that bisected the prefrontal cortex into superior and inferior regions (Alexander-Bloch et al., 2014). Although similarities in network properties of maturational and functional networks have been shown (Alexander-Bloch et al., 2013b), recent results (Alexander-Bloch et al., 2014), together with those reported here, suggest that the spatial distribution of maturational structural covariance networks may not map directly onto canonical functional networks. Further work is required to carefully characterize the relationship between developmental gray matter networks and brain systems defined based on functional connectivity.

Our results suggest a period of particular cortical thinning in late childhood in the anterior and posterior gray matter clusters that consist of more cognitive frontal, parietal, insular, and temporal regions. This has also been noted as a period of nonlinear change in cortical thickness networks, when local efficiency is reduced but global efficiency increases (Khundrakpam et al., 2013). In terms of cognitive development, late childhood corresponds to a period of rapid maturation of attention and executive functions (Pennington and Groisser, 1991; Hommel et al., 2004; Zhan et al., 2011). Our data suggest that cortical thinning most prominently in parietal regions, but also in the more anterior cortical network, may play a role in this process. A goal for future longitudinal studies will be to consider the maturational trajectories of gray and white matter networks in relation to cognitive maturation.

There are several limitations associated with this study. We used a single time point rather than longitudinal measurements to identify age slopes. Male and female participants were included in our analysis in similar numbers, though previous studies have shown evidence for sexually dimorphic trajectories in gray and white matter development (Giedd et al., 1997; Lenroot et al., 2007; Raznahan et al., 2011a). VBM analyses do not allow for separation of volume into thickness and surface area components, which make distinct contributions to volume development (Raznahan et al., 2011a). The age windows used here were defined to correspond with recent reports (Zielinski et al., 2010; Khundrakpam et al., 2013); however, these are somewhat arbitrary divisions and may not reflect optimal boundaries for transitions in age slope. The “optimal” number of clusters as defined using silhouette values did not match for gray and white matter clusters; the gray matter solution peaked at two clusters, separating cortical from cerebellar regions, while the white matter solution peaked at four clusters. We chose to model gray matter using a four-cluster solution, to match the white optimal white matter solution, and therefore note that gray matter clusters in the cortex may reflect gradations of a largely similar age-related volume pattern rather than substantially distinct clusters. Significant effects of data acquisition site and resolution were most prominent around the internal capsule, putamen, and posterior insula; clustering results in these areas may therefore be less reliable. Finally, we note that our analysis identified a set of gray and white matter clusters that covered regions that are very different in terms of cellular composition. As such, this analysis did not perform well at identifying regions with particular properties.

In summary, this work describes a correspondence between clusters of white and gray matter regions, defined in terms of age-related variability in volume across childhood and adolescence. We found that white matter voxels clustered together based largely on fiber direction, and gray matter regions divided into anterior, posterior, sensorimotor, and cerebellar clusters. These gray and white matter clusters could, nonetheless, be uniquely matched on the basis of spatial proximity, and showed parallel trajectories in age-related variability. This study identifies a previously unreported property of directional selectivity in white matter volume development, and demonstrates that white and gray matter volume clusters are linked across childhood and adolescence. There is a growing interest in understanding the role of anatomical networks in neurodevelopmental (Zielinski et al., 2012), neuropsychiatric (Alexander-Bloch et al., 2014), and neurodegenerative (Douaud et al., 2014) disorders; these results lay a foundation for studying network-level abnormalities in white matter volume and their relationship to gray matter covariance networks.

**Table:** Statistical table

	Data structure	Type of test	Power
a	White matter volume (normally distributed)	*K*-means clustering, silhouette values	n/a
b	Gray matter volume (normally distributed)	*K*-means clustering, silhouette values	n/a

Mean, SD, and range were calculated for age and IQ in each group. Handedness reflects right (R) versus left (L) hand preference. Mean adjusted income is measured in thousands of dollars.

## Figures and Tables

**Figure:**
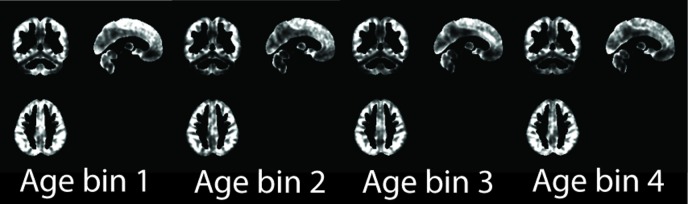


## Synthesis

The decision was a result of the Reviewing Editor Menno Witter and the peer reviewers coming together and discussing their recommendations until a consensus was reached. A fact-based synthesis statement explaining their decision and outlining what is needed to prepare a revision is listed below. The following reviewer(s) agreed to reveal their identity: Budhachandra Khundrakpam

The goal of the present study was to determine the covariance of white matter and gray matter volumes across childhood and adolescence in a large population sample from the NIH MRI Study of Normal Brain Development (360 kids 4 to 18 years of age). The use of such an extensive cohort is certainly a strength of the paper.

Although structural covariance of gray matter volume (or cortical thickness) has been shown in healthy and diseased brains, and in development and degeneration, the relation to covariance of white matter volume is unknown. This is an important issue that should be discussed more thoroughly in terms of the interpretation of the covariance patterns of white matter volumes and its relation to gray matter covariance. For example, structural covariance of gray matter volumes (or cortical thickness) has been hypothesized to be driven by coordinated maturational changes in cortical regions. What are the possible biological bases for the white matter covariance? The authors could also include some speculations on the relation between white and gray matter covariance.

Did the authors consider investigating separately for boys and girls? This will reduce the sample size for each time bin, but would give a valuable insight into sexual dimorphism of covariance patterns of white and gray matter volumes. A solution for this may be to look at only two time bins for this case.

The authors describe a number of clusters or networks in the brain based on their analysis. It is not clear how a cluster can comprise very dissimilar regions. For example the cluster associated with the cerebellum includes: the white matter within cerebellum, part of the cerebellar peduncles and the pons. The textures of these three compartments are vastly different. How can they be one cluster? These 3 components should have a completely different fiber orientation and composition (proportion of white matter and white matter + gray matter). That is, the analysis presented is not very discriminative. Furthermore, it might be of interest to discuss how the clusters reported here relate to functional networks in the brain that have been described by other methods such as for example fMRI. In relation to this, the authors may want to consider to change the usage of the term networks. Formally, the term network refers to the explicit description of the (anatomical or functional) relationship between at least two pairs of regions. In this context, a region of gray matter physically connected (white matter) to a second region of gray matter. That is, talking about gray matter networks and white matter networks suggest segregation between them rather than complementarity. Instead of referring to “networks” (since these were not formally assessed), the authors may refer to gray matter regions and white matter bundles or something like that.

In addition there are a number of serious methodological issues and other details that need to be addressed when submitting a revised version, in order to make the paper acceptable for publication.

1. MRI data was obtained from 6 different sites obtained at 2 different resolutions. Yet, authors do not report testing if there were any differences in results obtained at the two resolutions. This point could be critical sp. in the cluster analysis. Also was site controlled in the analyses? If so, please mention in the Methods.

2. Gray and white matter segmentation: All the analysis done is based on the reliability of a proper segmentation between gray and white matter. Yet, during development, contrast in T1-w images varies across ages. Did authors determine the reliability of the segmentation? Did they assess the distribution of variance of the signal? If contrast is reduced in the youngest kids, then there will be a bias from the beginning of the analysis. Furthermore, these issues with contrast are regional (vs. systemic). Was this potential source of error determined?

3. Clustering: There are several reports suggesting that cluster size distribution based on VBM depends on the local smoothness of the signal. Hence, it would be very useful if authors provided the local smoothness (RPV provided by SPM) of their data for each developmental group. Are they different? The results on the clusters depicted in the figures demonstrate large smoothing of data. That is, the topology of the clusters (specially in the gray matter) goes beyond the gray matter borders. Also, a discussion on the effects of not using the optimal clustering for all groups should be added.

4. Directional bias was based on the ICBM-DTI-81 atlas. This analysis is not too clear because authors do not describe how the dominant direction was assessed. In addition, the atlas has very limited number of pathways hence, the true direction was not really assessed. Why authors did not try to compute actual anisotropy (ex. via 3-d anisotropy histograms and gradient angle co-occurrence matrices) as others have done?

5. Indicate the standard deviations in the data.

6. Why did authors not report the volumetric data regressed by total brain volume? It seems an important variable to take into consideration during development.

7. Authors report on the slopes on volumetric values. Yet no description of how the slope was quantified and then statistically tested is presented in the current version.

8. The notion suggested by this work is that while white matter volumes increase, gray matter volumes decrease (that is, there is a gray matter thinning). This is hard to understand since the gray matter also contains multiple axons that in theory are in development. Can their result be related to the different MRI contrast alluded above producing a bias in the segmentation on younger cases?

9. The title of the manuscript implies the assessment of a “structural covariance” yet the manuscript does not describe any formal assessment of covariance.

10. Please check the spelling throughout for consistency. E.g. at some places, 'gray' is used and at others, 'grey'.

11. In the last sentence, please remove 'the'

Response to reviewer comments

We thank the reviewers for taking the time to provide detailed comments and helpful feedback. As detailed below, we have added several new analyses and made important clarifications to the manuscript.

1. Although structural covariance of gray matter volume (or cortical thickness) has been shown in healthy and diseased brains, and in development and degeneration, the relation to covariance of white matter volume is unknown. This is an important issue that should be discussed more thoroughly in terms of the interpretation of the covariance patterns of white matter volumes and its relation to gray matter covariance. For example, structural covariance of gray matter volumes (or cortical thickness) has been hypothesized to be driven by coordinated maturational changes in cortical regions. What are the possible biological bases for the white matter covariance? The authors could also include some speculations on the relation between white and gray matter covariance.

We have added the following paragraph to the Discussion to elaborate on this topic:

White matter development across childhood and adolescence is characterized by increased FA and volume, and reduced MD (Barnea-Goraly et al., 2005; Ben Bashat et al., 2005; Giorgio et al., 2008; Lebel and Beaulieu, 2011). These processes occur asynchronously across white matter regions (Lebel et al., 2008), and are believed to reflect changes in myelination and axonal packing (Beaulieu, 2002; Yakovlev and Lecours, 1967). White matter properties such as FA appear to be influenced by both genetic (Kochunov et al., 2015) and environmental (Hofstetter et al., 2013) factors. We speculate that genes expressed within regions or networks may contribute to coordinated patterns of volume development observed here. Though often considered separately, gray and white matter development may reflect processes occurring in the same cells (though changes in gray matter may also reflect changes in glial cells or vasculature (Zatorre et al., 2012)). We further speculate that coordinated white matter expansion and gray matter contraction may reflect a synergistic process of increased myelination and decreased synaptic or dendritic density that occurs as networks mature. We note however that MRI studies are quite limited in resolution and thus have limited ability to test these hypotheses directly.

2. Did the authors consider investigating separately for boys and girls? This will reduce the sample size for each time bin, but would give a valuable insight into sexual dimorphism of covariance patterns of white and gray matter volumes. A solution for this may be to look at only two time bins for this case.

This is a very interesting suggestion and we did look at boys and girls separately in our sample. We note that clusters were similar but noisier, likely due to smaller sample sizes. Unfortunately, however, the overall analysis strategy we adopted for this study was not designed to assess significant differences between groups. We could report qualitative differences, but do not feel that this would be particularly meaningful. This is an interesting question for future study with an analytic approach more appropriate to this question.

3. The authors describe a number of clusters or networks in the brain based on their analysis. It is not clear how a cluster can comprise very dissimilar regions. For example the cluster associated with the cerebellum includes: the white matter within cerebellum, part of the cerebellar peduncles and the pons. The textures of these three compartments are vastly different. How can they be one cluster? These 3 components should have a completely different fiber orientation and composition (proportion of white matter and white matter + gray matter). That is, the analysis presented is not very discriminative.

We do not disagree with the assessment that the analysis described here is not ideal for discriminating between regions with different fiber orientation and gray / white segmentation. However, we note that this was not the primary goal of the study. Rather we aimed to group regions that show a similar relationship with age. However, we have added the following to the limitations paragraph in the Discussion:

Finally, we note that our analysis identified a set of gray and white matter clusters that covered regions which are very different in terms of cellular composition. As such, this analysis did not perform well at identifying regions with particular properties.

4. Furthermore, it might be of interest to discuss how the clusters reported here relate to functional networks in the brain that have been described by other methods such as for example fMRI.

We have now expanded this paragraph in the Discussion:

Different distributed networks have been identified in functional MRI connectivity studies, including default-mode or task-negative, fronto-parietal / dorsal attention / task positive, ventral attention, salience, visual, motor and sub-cortical networks (Fox et al., 2005; Power et al., 2011). A previous, seed-based, study showed that longitudinal change in cortical thickness in core regions of the task-positive and task-negative networks are correlated (Raznahan et al., 2011). We note that using a data-driven approach, the gray matter clusters identified here did not specifically resemble either of these two networks. Instead, we found a posterior cluster that included both precuneus and bilateral intraparietal sulcus, regions associated with both the task positive and negative networks. An anterior cluster included both medial prefrontal task negative regions, as well as cingulate and dorsal prefrontal regions associated with task positive and salience networks. A recent study using a qualitatively similar approach to that described here also identified one primarily parietal network and several networks that bisected the prefrontal cortex into superior and inferior regions (Alexander-Bloch et al., 2014). Although similarities in network properties of maturational and functional networks have been shown (Alexander-Bloch et al., 2013), recent results (Alexander-Bloch et al., 2014), together with those reported here, suggest that the spatial distribution of maturational structural covariance networks may not map directly onto canonical functional networks. Further work is required to carefully characterize the relationship between developmental gray matter networks and brain systems defined based on functional connectivity.

5. In relation to this, the authors may want to consider to change the usage of the term networks. Formally, the term network refers to the explicit description of the (anatomical or functional) relationship between at least two pairs of regions. In this context, a region of gray matter physically connected (white matter) to a second region of gray matter. That is, talking about gray matter networks and white matter networks suggest segregation between them rather than complementarity. Instead of referring to “networks” (since these were not formally assessed), the authors may refer to gray matter regions and white matter bundles or something like that.

Throughout the manuscript when describing our results we now refer to 'clusters' instead of 'networks', and we agree that this is more appropriate. However, we have kept the term 'network' when situating our findings within the broader context of brain networks derived from functional, diffusion or structural covariance analyses. Our analysis has identified sets of regions with a shared property: their relationship with age. Conceptually this is not so different from analyses of inter-regional similarity in cortical thickness which have been described as 'structural covariance networks'.

6. MRI data was obtained from 6 different sites obtained at 2 different resolutions. Yet, authors do not report testing if there were any differences in results obtained at the two resolutions. This point could be critical sp. in the cluster analysis. Also was site controlled in the analyses? If so, please mention in the Methods.

While site and resolution were not accounted for in the previous draft, we have now repeated all analyses using models that include site and resolution as nuisance covariates. This has been clarified in the Methods:

Two general linear models were estimated in each age group, modeling a linear effect of mean-centered age on gray and white matter volume separately. Models included effects of gender, site (one regressor per site), and a linear effect of image resolution.

We have also run additional analyses to identify regions where volume may be particularly affected by these confounds. These analyses are described in the Methods:

Effects of site and resolution on VBM segmentation

As previous studies have shown that VBM segmentations may be affected by data collection site and acquisition parameters (Focke et al., 2011; Pardoe et al., 2008; Pereira et al., 2008; Takao et al., 2013), additional analyses were run to investigate effects of data collection site and image resolution. We note that participant age did not significantly vary by site (F(1, 360)=0.02, p=0.88). Resolution did show a significant negative trend with age as the youngest participants were more likely to have larger voxel size (F(1, 360)=12.6, p&lt;0.001). However, resolution did not significantly vary with age in individual age bins, though a trend remained in the youngest bin (p=0.051, p=0.73, p=0.60, p-0.22). To determine which regions may be affected by these parameters, models were run for gray and white matter volume separately including all 360 participants; effects of age, resolution, gender and site were modeled (one column per site). Fcontrasts were used to identify regions showing linear effects of resolution on gray and white matter volume, and non-linear effects of site. We then compared clustering results for models that included these covariates to a set of models that did not include site and resolution covariates, to assess effects of these parameters on clustering results.

And in the Results:

Effects of data collection site and resolution

Previous studies have noted that VBM estimates of gray and white matter volume are sensitive to differences in MR scanner and image resolution (Focke et al., 2011; Pardoe et al., 2008; Pereira et al., 2008; Takao et al., 2013). As the present study made use of a multi-site dataset, additional analyses were run to estimate potential impact of these factors on our results. A general linear model was estimated using the entire sample of 360 participants, including a linear effect of resolution and separate regressors modeling effects of each site. F-contrasts were used to identify regions sensitive to these effects. Results of F-contrasts for site are shown in Figure 6ab, thresholded at p&lt;0.001 uncorrected, for gray and white matter, respectively. We observe significant effects of site around the posterior putamen, orbitofrontal, inferior temporal and peripheral gray matter in Figure 6a. Significant effects on white matter volume were most prominent around the internal capsule (Figure 6b). Results of F-contrasts for resolution are shown in Figure 6cd, thresholded at p&lt;0.001 uncorrected, for gray and white matter, respectively. Affected gray matter regions were similarly concentrated around the posterior putamen and insula, occipital and dorsal prefrontal regions (Figure 6c). For white matter, similar to effects of site, effects of resolution were largely concentrated around the internal capsule (Figure 6d). We next compared clustering results for age β-values from models that did and did not include effects of site and resolution. These results are shown in Figure 6ef. We note that clustering results were largely similar between these two models. Figure 6gh show gray and white matter clusters obtained from these two models overlaid, regions of overlap are shown in purple. The only regions where cluster assignment substantially differed was around the putamen and internal capsule. Overall these results suggest that effects of site and resolution may have a fairly localized effect in sub-cortical regions, and we note that reliability of cluster assignment in these regions is a limitation of the present work.

7. Gray and white matter segmentation: All the analysis done is based on the reliability of a proper segmentation between gray and white matter. Yet, during development, contrast in T1-w images varies across ages. Did authors determine the reliability of the segmentation? Did they assess the distribution of variance of the signal? If contrast is reduced in the youngest kids, then there will be a bias from the beginning of the analysis. Furthermore, these issues with contrast are regional (vs. systemic). Was this potential source of error determined?

We have now clarified that reliability of segmentation was ascertained using visual inspection and an additional step was take to identify potential outliers:

VBM tools were also used to identify potential outliers by calculating the squared distance to sample mean in each age bin; no outliers were identified in this step.

We have also added an analysis of contrast variance as a function of age and site. We note that clustering analyses were performed on effects of age estimated within relatively narrow age windows (e.g. 5-8), and contrast is therefore less likely to have an effect. We also note that effects of age on image contrast have been most prominently described in the first few years of life and may be less pronounced in the age range studied here, i.e. 5-18 years. Indeed, results of the contrast analysis showed that age did not have a significant effect on contrast across any of the regions we investigated.

These are described in new sections in the Methods:

Effects of age on image contrast T1-image contrast is known to increase over the first few years of childhood (Paus et al., 2001; Shi et al., 2010). Although the population assessed here was older (i.e. the youngest participant was 4.8y), systematic effects of image contrast may nonetheless contribute to variable quality of gray/white segmentation, and these effects may vary between brain regions. To assess effects of image contrast as a function of age, bilateral masks covering frontal, temporal, parietal and occipital gray and white matter regions were generated using the Wake Forest Pick Atlas tool (Maldjian et al., 2003) and the TD-ICBM-152 atlas (Mazziotta et al., 2001). These masks were warped into each participant's native space and used to extract regional gray and white matter values. Contrast was calculated in each subject and each region as: C= (White matter intensity -Gray matter intensity)/ Gray matter intensity. We then assessed effects of age and site on contrast across the sample and within each age bin.

And in the Results:

Effects of image contrast

White/gray matter contrast values for each lobe (frontal, temporal, parietal, occipital) were entered into ANOVAs modeling effect of site with age as a covariate. Models were run across the entire sample, as well as within each age bin. This analysis showed a significant effect of site in the temporal (F(1,357)=16.6, p&lt;0.001) lobe, and a trend level effect in the frontal lobe (F(1,357)=4.3, p=0.04 uncorrected for multiple comparisons). Over the entire sample there was a trend-level, negative, association with age in the parietal cortex (F(1,357)=3.9, p=0.048 uncorrected). However, age was not a significant predictor of contrast within any of the age bins, for any of the lobes. From these results we conclude that image contrast is unlikely to have biased age-slopes in this analysis.

8. Clustering: There are several reports suggesting that cluster size distribution based on VBM depends on the local smoothness of the signal. Hence, it would be very useful if authors provided the local smoothness (RPV provided by SPM) of their data for each developmental group. Are they different? The results on the clusters depicted in the figures demonstrate large smoothing of data. That is, the topology of the clusters (especially in the gray matter) goes beyond the gray matter borders.

We believe that the reports the reviewer is referring to describe differences in cluster-level significance depending on the amount of smoothing applied (e.g. Silver et al. Neuroimage 2011). We note that this is somewhat different than the clusters we report, which are simply sets of voxels grouped together by kmeans based on similar age effects. We include here images of local smoothness (i.e. SPM RPV maps) in each age group. These are largely similar across age groups, but not identical. We are unsure how to incorporate this information into the manuscript and would be grateful if the reviewer could provide additional guidance on this issue.

9. Also, a discussion on the effects of not using the optimal clustering for all groups should be added.

We have added the following to the limitations paragraph in the Discussion:

The 'optimal' number of clusters as defined using silhouette values did not match for gray and white matter clusters; the gray matter solution peaked at two clusters, separating cortical from cerebellar regions, while the white matter solution peaked at four clusters. We chose to model gray matter using a four-cluster solution, to match the white optimal white matter solution, and therefore note that gray matter clusters in the cortex may reflect gradations of a largely similar age-related volume pattern rather than substantially distinct clusters.

10. Directional bias was based on the ICBM-DTI-81 atlas. This analysis is not too clear because authors do not describe how the dominant direction was assessed. In addition, the atlas has very limited number of pathways hence, the true direction was not really assessed. Why authors did not try to compute actual anisotropy (ex. via 3-d anisotropy histograms and gradient angle co-occurrence matrices) as others have done?

The goal of this analysis was to gain a general picture of directional bias in each white matter cluster, rather than to obtain the precise direction at each voxel. While we agree that the ICBM-DTI atlas is limited, we feel that it is adequate for this purpose. Note that direction was not computed based on the tracts delineated in the ICBM-DTI-81 atlas, but rather by using the eigenvector information at each voxel. We have now attempted to clarify the steps of this analysis and have added Figure 5 to illustrate the results. From the revised section of the Methods:

Directional bias in white matter clusters

Visual inspection of white matter clusters indicated a potential directional bias. To test this, white matter clusters were compared against directional maps from the ICBM-DTI-81 Atlas (Mori et al., 2008) to determine the primary direction of white matter fibers in each cluster. This atlas includes estimated eigenvalues for eigenvectors corresponding to three principal directions (x:right-left, y:anterior-posterior, z:superior-inferior). To determine whether voxels assigned to each white matter cluster had a preferred direction, the three eigenvalue maps were masked by each white matter cluster. For each cluster the subset of voxels with a dominant orientation along one of these principal directions was obtained by thresholding to include only voxels for which at least one eigenvalue {greater than or equal to} 0.4. We then calculated the proportion of voxels for which the maximum eigenvalue was in each principal direction.

11. Indicate the standard deviations in the data.

We have now added standard deviations to the cluster centers panels of Figures 1-4.

12. Why did authors not report the volumetric data regressed by total brain volume? It seems an important variable to take into consideration during development.

We have now elaborated on our rationale for not modeling effects of total tissue volume in the main analysis, and added clustering results from models using proportional scaling and ANCOVA to account for effects of tissue volume (Figure 7). These are now described in the Methods:

Effects of modeling total gray and white matter volume

Many VBM studies model effects of total tissue volume in VBM, enabling the identification of regions that discriminate between groups after differences in total volume are accounted for (Peelle et al., 2012). Significance of regional effects of age are sensitive to the choice of model (Peelle et al., 2012). For the main analysis here, we chose not to account for total gray and white matter volume, as our goal was simply to model age trends and not to identify regions where age effects were greater than the mean. However, to investigate differences in our results when accounting for total volume, two additional models were run, using proportional scaling by total tissue volume and including total tissue volume as a nuisance covariate.

And in the Results:

Effects of modeling total gray and white matter volume

Two additional analyses were run using proportional scaling by total tissue volume and including total tissue volume as a covariate (ANCOVA). The resulting parameter estimates for age were entered into a similar cluster analysis as that described above. These analyses identified very similar clusters (Figure 7), however with some differences specifically in white matter clustering in the midbrain for the ANCOVA model.

13. Authors report on the slopes on volumetric values. Yet no description of how the slope was quantified and then statistically tested is presented in the current version.

This point has now been clarified in the Methods:

Clustering based on gray and white matter age-slope

For each tissue type (white and gray matter), parameter estimates for the effect of age in each of the four age bins were obtained for each voxel. All parameter estimates (β values) were entered into a pair of matrices, one gray and one white matter, in which each row corresponded to a voxel and each column corresponded to an age-bin. Analyses were performed on all voxels independent of the significance of age effects, that is, voxel-level significance of age-effects was not assessed as part of this study. Matrices were entered into k-means clustering in MATLAB to identify clusters of voxels with similar age-slopes across these developmental stages.

14. The notion suggested by this work is that while white matter volumes increase, gray matter volumes decrease (that is, there is a gray matter thinning). This is hard to understand since the gray matter also contains multiple axons that in theory are in development. Can their result be related to the different MRI contrast alluded above producing a bias in the segmentation on younger cases?

Gray matter thinning and white matter volume expansion across late childhood and adolescence have been described in a number of developmental MRI studies (e.g. (Giedd et al., 1999; Gogtay et al., 2004; Lebel and Beaulieu, 2011; Shaw et al., 2008; Sowell et al., 2004)). Unfortunately, due to the resolution and contrast available from MRI, it is difficult to ascertain the cellular mechanisms underlying these changes. The question of whether proliferation of myelin in gray matter contributes to developmental changes has been discussed in other studies (Shaw et al., 2008; Sowell et al., 2004). A recent study suggested that development of superficial white matter and cortical thinning were not strongly associated, which speaks against this theory (Wu et al., 2014). Unfortunately these are not effects that we can reasonably ascertain with the data presented here. However, as noted above, we did not find reduced GM / WM contrast in our youngest participants, and therefore do not believe that the results described here were biased by this effect.

15. The title of the manuscript implies the assessment of a “structural covariance” yet the manuscript does not describe any formal assessment of covariance.

The title has been edited to: Synergistic effects of age on patterns of white and gray matter volume across childhood and adolescence

16. Please check the spelling throughout for consistency. E.g. at some places, 'gray' is used and at others, 'grey'.

All instances of 'grey' outside of the references have now been changed to 'gray'.

17. In the last sentence, please remove 'the'.

This has been done.
